# Respectful care for postpartum women with sickle cell disease: a netnographic study

**DOI:** 10.1590/0034-7167-2023-0545

**Published:** 2024-12-16

**Authors:** Zannety Conceição Silva do Nascimento Souza, Luana Gabriella Pinheiro Barrêto, Paulo Roberto Lima Falcão do Vale, Elysangela Dittz Duarte, Cristiane dos Santos Silva, Evanilda Souza de Santana Carvalho

**Affiliations:** I Universidade Estadual de Feira de Santana. Feira de Santana, Bahia, Brazil; II Hospital Unimed Baía de Todos os Santos. Feira de Santana, Bahia, Brazil; III Universidade Federal do Recôncavo da Bahia. Santo Antônio de Jesus, Bahia, Brazil; IV Universidade Federal de Minas Gerais. Belo Horizonte, Minas Gerais, Brazil

**Keywords:** Pregnant Women, Anemia, Sickle Cell, Care, Obstetric Nursing, Culturally Competent Care., Mujeres Embarazadas, Anemia de Células Falciformes, Atención, Enfermería Obstétrica, Asistencia Sanitaria Culturalmente Competente.

## Abstract

**Objectives:**

to analyze principles of respectful maternity care in narratives of postpartum women with sickle cell disease, relating them to Sustainable Development Goals.

**Methods:**

netnographic study, with two videos published in 2020. Deductive iconographic and thematic analysis by Respectful Maternity Care Charter, organized in MAXQDA.

**Results:**

principles identified were the right to: freedom from harm and ill-treatment; information, informed consent, refusal of medical procedures, and respect for their choices and preferences including companion; be considered a person from birth, with dignified and respectful treatment; health at the highest possible level; newborns being with their parents or guardians. The Sustainable Development Goals for women by 2030 were not positively contemplated in postpartum women’s experience.

**Final Considerations:**

it is appropriate that health workers qualify themselves to provide respectful maternity care, with qualified listening, understanding, and resolution of unique demands of postpartum women with sickle cell disease, seeking equality in care for women.

## RESUMEN

**Objetivos:**

analizar los principios del cuidado materno respetuoso en narrativas de gestantes con anemia falciforme, relacionándolos con los Objetivos de Desarrollo Sostenible.

**Métodos:**

estudio netnográfico, con dos videos publicados en 2020. Análisis iconográfico y temático deductivo del *Respectful Maternity Care Charter*, organizado en el *software* MAXQDA.

**Resultados:**

los principios identificados fueron el derecho a: estar libre de daño y maltrato; información, consentimiento informado, negativa a procedimientos médicos, respeto a las elecciones y preferencias de las mujeres, incluidas sus acompañantes; ser considerado una persona desde su nacimiento, con trato digno y respetuoso; salud al más alto nivel posible; recién nacido esté con sus padres o tutores. Los Objetivos de Desarrollo Sostenible hasta 2030 para las mujeres no se reflejaron positivamente en la experiencia de las mujeres en el mundo laboral.

**Consideraciones Finales:**

es oportuno que los trabajadores de la salud estén capacitados para brindar una atención materna respetuosa, con escucha calificada, comprensión y resolución de las demandas singulares de las parturientas con anemia falciforme, buscando la igualdad en la atención a las mujeres.

## INTRODUCTION

Care for women with sickle cell disease (SCD) during pregnancy, childbirth and the postpartum period requires visibility because it is a genetic and hereditary disease, which is estimated to affect 60,000 to 100,000 people in Brazil. SCD is clinically and epidemiologically relevant, with a high prevalence and morbidity and mortality, especially in the black Brazilian population, and is therefore considered an important public health concern in Brazil^(1,2)^. In Brazil, the highest incidence is found in the states of Bahia, Distrito Federal and Piauí^(3)^.

In 2001, through Ordinance 822 of the Ministry of Health (MoH), neonatal screening for SCD and other hemoglobinopathies was instituted through the heel prick test in the Brazilian National Neonatal Screening Program (PNTN). In 2011, the MoH included hemoglobin electrophoresis test in prenatal routine for pregnant women and partners^(3)^. These initiatives demonstrate an understanding of the severity of SCD and the importance of its early detection.

Between 2014 and 2020, there was an annual average of 1,087 new cases of children diagnosed through neonatal screening, with an incidence of 3.78 per 10,000 live births. Regarding mortality, data from the *Sistema de Informações de Mortalidade* (SIM, Mortality Information System) related to the years 2014 and 2019 show a higher number of deaths in the age group between 20 and 29 years old. Among children aged 0 to 5 years old, there is an average of one death per week^(3)^. These data show that it is necessary to give visibility to this public health concern. Although it is not exclusive to the black population, the majority of those affected are concentrated in this population, and due to the greater social vulnerability of this group, the highest mortality rate due to the disease is also recorded^(1-4)^.

SCD interferes with women’s sociability, the exercise of their sexuality, reproductive choices, marital relationships, motherhood plans, interpersonal relationships and self-image^(5)^. Furthermore, women with SCD have different expectations about their pregnancy and birth than women without the disease. Since pregnancy is considered high-risk, it requires frequent exams, strict control of lifestyle habits, involves insecurity about their own health and that of the fetus, fear of death and the imminent need to undergo a cesarean section^(6)^.

Inequalities of race, gender and class, education and income levels, and housing conditions increase the vulnerability of black women when compared to white women, interfering with access to services^(7,8)^ and, consequently, with disease prognosis. It is assumed that women with SCD have unique experiences of pregnancy, labor and delivery, determined by chronic illness, but also by racism and socio-racial inequities. Thus, racism is “a systematic form of discrimination that is based on race and manifests itself through conscious or unconscious practices that culminate in disadvantages or privileges for individuals depending on the racial group to which they belong”^(9)^.

Racism involves racial prejudice and discrimination and, consequently, discriminatory practices that result, among other things, in differential treatment for racialized groups. In view of this understanding, one of the ways to curb racism in the care provided to black women during pregnancy and childbirth is by promoting respectful care. Respectful maternal care promotion is mentioned as an element in improving maternity care strategies and includes meeting the fundamental rights of women, newborns and families through equitable access to care based on good practices^(10)^.

From this perspective, the global public health sector emphasizes the importance of understanding and discussing the harm experienced by women during childbirth in health services. In 2011, the White Ribbon Alliance published the Respectful Maternity Care (RMC) Charter: The Universal Rights of Women and Newborns, which has been implemented in many countries as an action of this global movement to defend safe motherhood as a priority for governments^(11)^. In 2014, the World Health Organization (WHO) released a statement entitled The prevention and elimination of disrespect and abuse during facility-based childbirth, using the RMC Charter as one of its references, reaffirming the fundamental human rights of women during childbirth and highlighting right to healthcare and to the highest attainable level of health, which includes the right to dignified and respectful health care^(12)^.

Supported by the rights of women and newborns in the context of pregnancy and childbirth, respectful maternity care is presented by the RMC Charter in nine principles to be contemplated during care: right to freedom from harm and ill-treatment; right to information, informed consent, and respect for their choices and preferences, including companion of choice during maternity care and refusal of medical procedures; right to privacy and confidentiality; right to be considered their own person from the moment of birth and has the right to be treated with dignity and respect; right to equality, freedom from discrimination and equitable care; right to healthcare and to the highest attainable level of health; right to liberty, autonomy, self-determination and freedom from arbitrary detention; child’s right to be with their parents or guardians; child’s right to an identity and nationality from birth; and the right to adequate food and clean water^(11)^.

To achieve safe childbirth and birth, public policies in Brazil have defined as guidelines equity, respect, care networks, adequate childbirth, prevention of maternal mortality, prenatal care, prevention of prematurity, literacy, empowerment and engagement, family and community participation^(13)^.

These guidelines as well as the RMC Charter align with the Sustainable Development Goals (SDGs) for 2030, proposed by the United Nations (UN). Two of these goals reflect on women’s health, when they propose to “ensure healthy lives and promote well-being for all at all ages” and “achieve gender equality and empower all women and girls”^(14)^.

In the face of global initiatives for respectful childbirth, discussing care for postpartum women with chronic diseases calls us to access available knowledge to break with social determination in health and to reflect on the care offered to black women in the pregnancy-puerperal cycle, since this group is more vulnerable to violence, negligence and violation of rights resulting from racism in health institutions.

Racism manifests itself in health institutions not only through prejudice and racial discrimination, but also through institutional racism (IR), which involves the construction and maintenance of systems of inequality based on individuals’ race/skin color, and is reproduced in institutions, whether public or private. It constitutes a dimension of racism in which people receive unequal treatment, with unequal results within health units, configuring a selective exclusion of groups considered racially subordinate. It is, therefore, an expression of racism that goes beyond the individual dimension and establishes itself as a structural dimension, through policies, practices, norms and forms of organization that perpetuate racist attitudes towards people who use health services^(15)^. Therefore, it is not possible to address black women’s health care without also discussing racism.

The health demands of black pregnant women with SCD still lack visibility in public policies and in discussions and scientific productions on respectful care during childbirth, since it is not clear what these women consider to be respectful care. Thus, this article was developed based on the following question: what are the principles of respectful maternal care identified in the narratives of postpartum women with SCD?

## OBJECTIVES

To analyze the principles of respectful maternal care in narratives of postpartum women with SCD, relating them to the SDGs.

## METHODS

### Ethical Aspects

Regarding ethical aspects, the principles of respect for privacy, honor, inviolability of the image and dignity of a person were considered in accordance with the General Law for the Protection of Personal Data (LGPD), Law 13,709/2018. It is worth noting that the videos on the digital platform are freely accessible to the public and, as a result, there was no direct contact with the channel owners. In addition to this, written consent from the owners is not required, since they, as data subjects, made the information public^(16)^. Respecting confidentiality and anonymity, the identity and image of postpartum women were omitted, and the links to selected videos were not revealed.

### Type of study

This is a netnographic study conducted on the YouTube^TM^ video platform. This qualitative research report was based on the COnsolidated criteria for REporting Qualitative research (COREQ)^(17)^. Netnography investigates social phenomena experienced on the internet through the use of electronic devices. It is possible to use digital communications, investigate interactions on social networks, blogs, forums, websites, seeking to interpret how netnographic culture is expressed^(18)^.

### Study setting

The digital platform YouTube^TM^, created in 2005, allows videos to be shared and viewed by millions of people who access the internet (hence the choice of this field for the study), being an important vehicle for information, and favors interaction among those who watch through comments, likes and dislikes^(19)^.

### Data Source

The two selected videos address reports on childbirth experiences of two women living with SCD and have some characteristics: video 1 - produced in 2020, 7 months after the rapporteur’s birth, it lasts 35 minutes and 3 seconds, with 589 views until April 18, 2023; video 2 - also produced in 2020, 3 months after the rapporteur’s birth, it lasts 5 minutes and 47 seconds, with 2,200 views until April 18, 2023. The names of both videos have been preserved for ethical reasons.

### Data collection and organization

The first stage of data collection and organization consisted of finding out what women had to say about childbirth in the context of SCD. In the second stage, we searched and selected videos on YouTube^TM^, including those that addressed childbirth experiences experienced by women with “SCD” or “sickle cell anemia”, with no posting date limit, in Brazilian Portuguese, containing experiences narrated by the women themselves. Videos with derogatory, entertainment-related or dubious content (fake news) were excluded. To verify the veracity of the videos’ content, we used SCAN^(20)^.

**Figure 1:**
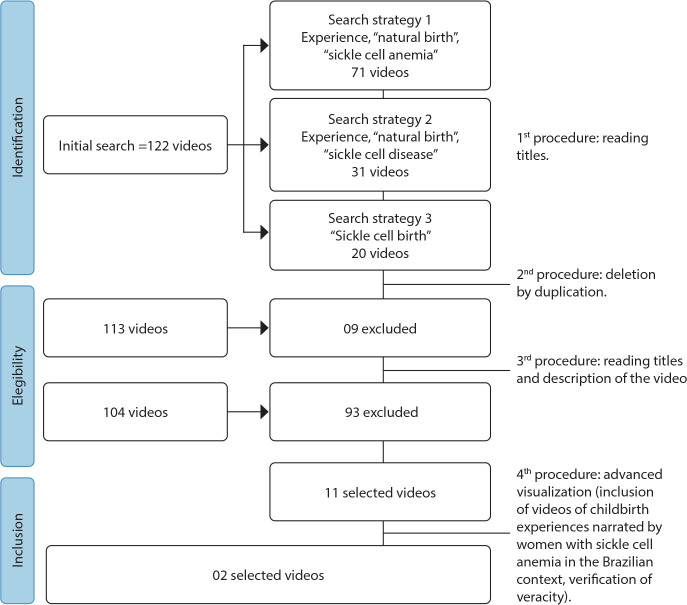
*Corpus* constitution adapted from the PRISMA proposal^(21)^

To construct the search strategy, authors 1, 3 and 6 developed the protocol with the following keywords (tags): experience; “normal birth”; “sickle cell anemia”. Then, a pilot search was carried out in the YouTube™ search box. One of the tags was “normal birth”, as we realized that it would lead us to videos about births, but this was not necessarily an inclusion criterion, since cesarean section is common in women with SCD. To avoid bias in video selection, this stage was performed blindly by authors 1, 2 and 3 (qualified in the method). In the absence of consensus, author 6 (expert in the method and subject) contributed to the final decision. The data were organized in a Microsoft Word^®^ version 16 document. The complete data collection protocol, which includes the identification, eligibility and inclusion stages, in compliance with the Preferred Reporting Items for Systematic Reviews and Meta-Analyses (PRISMA) flowchart^(21)^, is illustrated in Figure 1.

Video sampling was intentional, being selected for the wealth of information sought, involving reflexivity and in-depth understanding of subjective meanings in that cultural context^(22)^.

In the third stage, the selected videos were watched, and women’s statements were transcribed in full by a fourth independent researcher (author 2). Transcription was validated by author 1. In the fourth stage, the transcribed text was organized in Microsoft Word^®^ into individual files and inserted into MAXQDA version 20 (reference number 282394887). In the fifth stage, an iconographic analysis plan was constructed.

In the sixth stage, the list of codes was organized with a dictionary of meanings supported by the RMC Charter^(11)^, which was discussed and agreed upon by the research team to guide coding.

In the seventh stage, coding was carried out by author 1, who had experience in the subject, and by author 4, who had expertise in qualitative research. Aiming at reliability in the analysis, coding was carried out independently by authors 1 and 2, guided by the code list and with support from MAXQDA. Also in this phase, in parallel with peer coding, iconographic analysis of the videos took place independently by author 2 and author 3, and subsequent validation by the entire research team. Thus, collection and analysis followed a process of triangulation of researchers.

In the eighth stage, a narrative summary was prepared to describe the results found. In the ninth stage, triangulation of analyzed data was adopted, which was obtained through coding (themes assigned to transcripts) of excerpts of corresponding narratives and images, as described in Chart 1.

**Chart 1:** Respectful Maternity Care Charter principles, related narratives, and iconographic analysis of YouTube^TM^ video images

RMC Charter^*^ principle (code)	Selected narrative	Iconographic analysis	Inference
Right to freedom from harm and ill-treatment^▲^	*Ah... after all that mistake of hers, of not knowing what sickle cell anemia is and wanting to send me to the clinic... she made the second mistake... of not looking at my x-ray. I was hospitalized for a week, feeling very tired, you know... I cried because I couldn’t take it anymore... the... doctor saw how I was and said, “You have to hold on as long as you can... the longer your baby stays in there, the better it will be...”* (V1 starting minute 6’59’’ to 7’40”).	Description V1: 8”30’ Eyelids move apart, opening the eyes as wide as possible. Lips come together, leaving a small gap between them, with the upper dental arch closed over the lower. Corners of the mouth elongate, bringing them closer to the ears. Both hands touch the cheeks on each side of the face simultaneously. The third finger of each hand touches the tip of the eyebrow on its respective side, palmar region close to the chin.	Expressions of fear and apprehension when reporting the experience of medical care.
Child’s right to be with their parents or guardians^▲^	*... they asked, “Doctor, can you show her the baby?”, I don’t know why, you know? ... I don’t know if they really thought I wouldn’t survive... so, they showed her to me briefly, they put her in the incubator and took her to the neonatal ICU, because she was born prematurely... I don’t remember her features, how she was born... and she was born weighing two and a half kilos, now the centimeters... I don’t know to this day. I went back to the ICU, I couldn’t see her anymore... until after I could hold her in my arms... I could go home... I was discharged with her...* (V2 - starting minute 4’13” to 5’25’).	Description V2: 5’ 21” Head down, looking down towards the baby on her lap (only a small part of the baby’s scalp can be seen). Lips close together, cleft between them exposing the upper dental arch, contraction of the risorius and zygomatic muscles, expression lines on the cheeks.	Anguish over waiting for her daughter. Joy and satisfaction in being home with your daughter.
Right to be considered their own person from the moment of birth and has the right to be treated with dignity and respect^▲^	*Then I remember a doctor came in... ridiculous, you know? I wanted to know what medication I was taking, what I had, I wanted to know everything!... he took me, looked at my face and said, “Hey, you’re not here because you have pneumonia, you’re here because of your anemia!”* (makes an ironic expression) *... can you believe he said that to me? My anemia wasn’t low... pain... I wasn’t feeling anything like that about anemia... the only thing I was feeling was shortness of breath, tiredness, weakness, you know? And pain in my lungs, which I felt, so everything I was feeling was because of the pneumonia, and he looked at me like that and said it wasn’t because of the pneumonia, it was because of the anemia... so, wow! I wanted to punch that doctor in the face... he came to talk to me, “Your exams are all altered and you’re going to have to stay here for at least another seven days, because your exam showed that you have an... an infection that... is already in... your entire body.” He said to me, “And we don’t know what kind of infection it is, so you have to stay here so we can change your medicine to a much stronger one.” I was getting nervous and upset, because how was I going to stay away from my daughter for another seven days? Then I started to cry and started to give up...* (V1 - starting minute 26’13” up to 29’14”).	Description V1: 28’ 41” Woman brings her eyelids and eyebrows together tightly. The fingers of the hands come together in pairs in front of the face, lateral region of the index fingers, touching the space between the eyebrows. The palms of the hands are apart, and the 1^st^ finger points outwards from the face.	Nervousness, sadness, indignation, irritation and aggression towards the doctor for not answering her questions about her health clearly. Despair, malaise, loss of appetite, repercussions of poorly communicating difficult news and suffering due to being separated from her newborn daughter.
Right to information, informed consent, and respect for their choices and preferences, including companion of choice during maternity care and refusal of medical procedures^▲^	*... they said, “...we’ll see if we can schedule your delivery...for tomorrow!”. I had just woken up, hadn’t had breakfast yet, and then they came back to me and said, “Oh, we’re going to deliver your baby...”. So, I had to run, call my husband, call my mother. It wasn’t exactly at the time they said, it delayed the delivery a little. Anyway, they came to get me and took me to the operating room. Since my case was more serious, I had to have a cesarean section, I had no choice...* (V2 - minutes 0’47” to 1’57”).	Description V2: 1’ 33” Woman turns her head slightly to the left, turns her eyes to the left and upwards, contracts her lips and risorius muscle. Raises her left hand, with the palmar surface facing upwards and the five fingers apart from each other.	Ironic expression of surprise and dissatisfaction.
Right to healthcare and to the highest attainable level of health**^■^**	*... in the room, there was the team that was going to deliver my baby, obstetricians, doctors for my health problems, the ICU doctor, a hematologist, because I have sickle cell anemia. I really liked it, because they turned off the air conditioning, put a mattress under the stretcher to keep me warm, because anyone with sickle cell anemia knows what it’s like. There was an angel* (anesthesiologist)... *he was always telling me, “We’re going to do this procedure... this injection is for this thing...”. Since I was feeling very short of breath, he also helped me, he increased the air. When I was already okay and my saturation was okay, he lowered it a little more* (V2 - minute 1’33’’ to 2’34”).	Description V2: 1’ 33’’ Serious facial expression, gestures with hands while reporting. Blinks eyes a lot, moves head and torso gently during the report, smiles when saying that they turned off the air conditioning.	Relaxation and comfort by mentioning caring practices such as turning off the air conditioning.

*Caption:*

▲ *rights denied;* ^**■**^rights guaranteed;

* RMC - Respectful Maternity Care.

### Data analysis

For data analysis, iconographic analysis was used, which makes it possible to examine and interpret visual content based on the details selected in it. Moreover, it is possible to compare images, attributing a sense and meaning to the context, to the ways in which images and distinct groups relate, and discussing them with the support of texts, archives, interviews and the like^(23)^.

Iconographic analysis occurred in four moments^(24)^: 1) viewing selected videos in full, without audio and without subtitles, searching for expressions that were related to the phenomenon studied, with print; 2) viewing with pauses, without audio and subtitles, analysis of static images, guided by the following questions: who are the actors? What are the inanimate objects and other characteristics of the environments that make up each scene? 3) viewing the videos with pauses, audio and subtitles, with the objective of answering the following questions: what are the body expressions that stand out? What are the symbols, gestures and behaviors that illustrate the experiences lived? How do actors interact? What are the principles of respectful care identified?; 4) viewing the videos in full, without pauses, with audio and subtitles.

In thematic analysis, the elements that answered the research question, broad patterns of meaning, were observed. After reviewing the themes, the pattern of meanings was refined and defined. In the final writing of results and analysis, the narrative was related to extracted data and existing literature^(25)^. This analysis was thematically deductive, as it was guided by the theoretical interest in the RMC Charter principles, when analyzing women’s narratives. Thus, based on an existing theoretical construct, the phenomenon was analyzed in detail in the aspects of the data that were related to the principles mentioned^(25)^.

## RESULTS

## DISCUSSION

The narratives of women with SCD studied highlighted aspects of five principles advocated in the RMC Charter for respectful care in maternity, but most of them have weaknesses regarding the guarantee of rights. Moreover, it is possible to perceive that the established SDG by 2030 was not positively contemplated in the postpartum women’s experience.

Regarding the SDGs^(14)^, SDG 3 presents the goal to “Ensure healthy lives and promoting well-being for all at all ages”. Item 3.1 of this SDG recommends that by 2030 maternal mortality worldwide be reduced to less than 70 deaths per 1,000,000 live births, and item 3.2 addresses the need to end preventable deaths of newborns and children under 5 years of age (reduction of at least 25 per 1,000 live births), with all countries seeking to reduce neonatal mortality to at least 12 per 1,000 live births.

The errors and mistakes in health professionals’ skills in monitoring postpartum women with SCD, evidenced in their statements, weaken the achievement of this SDG, since professional health training that does not include content and practices for caring for women with this uniqueness can cause complications in these women’s and their fetuses’ health, increasing the risk of maternal, fetal or neonatal mortality.

The RMC Charter principles, such as the “right to freedom from harm and ill-treatment” and the “right to information”, were not fully guaranteed; there was guidance on the need to maintain pregnancy for as long as possible due to the risk to the fetus, but this information was not clear enough for women to understand that it was related to the risk of premature birth. As women with SCD expressed their pain resulting from the pathophysiology of their disease, professionals’ response was limited to telling them to endure the discomfort in order to maintain the pregnancy and benefit the fetus. Black women receive less guidance on pregnancy, childbirth, postpartum care, and emergency situations in prenatal consultations when compared to white women, and this information is still very restricted and fragmented, with less quality as the women’s level of education decreases^(26)^.

Although pregnancy is a potentially serious event for women with SCD, this condition does not constitute an impediment to having children. It is of utmost importance that, during pregnancy, women are properly guided by multidisciplinary team professionals, receiving information about the risks and possible complications of their pregnancy, birth and postpartum period.

Thus, one can reflect on what would be the causes of this low supply of information: non-humanitarian, mechanistic, productivist and interventionist training of health professionals; greater appreciation of technological interventions on human bodies; emotional neutrality in the relationship with the people served; and structural racism.

The vertical and unilateral relationship and the overvaluation of scientific knowledge and epistemicide of popular knowledge caused by health professionals restrict access, making it difficult to create bonds and, consequently, to provide care from a longitudinal perspective. These aspects violate item 3.7^(14)^, as it deals with ensuring universal access to sexual and reproductive health services, including family planning, information and education, which was denied by the rapporteurs’ statements.

The report in video 1 shows a lack of continuity in the care provided by the institution and the lack of preparedness of health professionals. In this context, regarding the care provided to pregnant women with SCD, the MoH points out that “health professionals are still very unprepared to provide care, particularly during pregnancy, which can contribute to increasing the insecurity and fear that women experience at this stage of life”^(6)^.

The lack of preparedness is expressed in dealing with gestational risk, in the lack of coordination between the various institutions that women with SCD seek care for, and in this research, specifically in the conduct, which demonstrates a greater concern with the restriction of fetal growth, which can induce premature birth, by the phrase “hold the pregnancy as long as possible”, and others with little understanding by women, such as “infection that is all over the body”, by the report of delay in diagnosing pneumonia, even after having undergone an X-ray, described in video 1. Pneumonia is another very common condition in people with SCD. Thus, the expression “highest attainable level of health” includes not only access to diagnostic equipment and services, but also assistance from qualified professionals.

Video 1 iconographic analysis demonstrates nervousness, sadness, indignation, irritation and aggression directed at the doctor for not answering women’s questions about their health status clearly. This calls for reflection on how little knowledge and professional unpreparedness to deal with SCD can contribute to dehumanized and disrespectful care that reproduces violence and violates the rights of black women with SCD, and this can also lead to negative outcomes for mothers’ and babies’ health.

The fact that women report inappropriate behavior and describe their questions to health professionals, sometimes in an ironic way, may give the impression that they have used empowerment. A study conducted with women in Nepal^(27)^ concluded that there was a significant association between women’s empowerment and the use of maternal health services. The study categorized empowerment into three elements ((1) media and information technology; (2) economic; and (3) sociocultural and family/interpersonal), with the first two being more influential than the last in this search for qualified services. For the researchers, in relation to women “... providing and encouraging them to become financially independent and access to media and information technology can increase the number of women seeking qualified maternal health services”.

However, when considering the care provided to black women with SCD during childbirth, who are vulnerable due to several issues already mentioned, with their birth being defined as a cesarean section, without the possibility of choice, this empowerment is still a desired goal that needs to be achieved through the actions of professionals, institutions and public policies. The power of health professionals is still imposed and culturally established in health services instead of women’s leading role, including through the training of professionals that feeds this power.

Achieving gender equality and empowering all women and girls, ending all forms of discrimination that make up SDG 5, crosses this situation in the childbirth of women with SCD, and is one of the most difficult goals to be achieved, since part of the culture of the power of scientific knowledge overrides women’s will, their knowledge and autonomy over their bodies. It is important to increase the use of information and communication technologies, with solid policies for empowerment.

Respectful care in maternity also includes newborns and their right to be with their parents or guardians. This care was observed in this study, with divergent points, such as: women reported that they were not introduced to their newborns immediately after birth, which raises concerns about the need for emergency medical assistance or whether there was a careless attitude in this regard.

It is important to consider that newborns’ context and clinical conditions may require intensive care that prevents mothers from seeing the baby immediately after birth. In situations like this, the team needs to explain the need for temporary separation between mother and newborn for immediate care and communicate therapeutic possibilities, reducing stress and anxiety and ensuring mother and child safety. Skin-to-skin contact seeks to encourage early contact between newborn and mother at birth and, whenever possible, should be performed in the delivery room, as it has many benefits for both: creating a bond; increasing newborns’ tranquility; reducing the loss of body temperature, the risk of hypoglycemia and length of hospital stay; encouraging breastfeeding and milk letdown in the first half hour of life; reducing mothers’ anxiety to confirm their child’s safety and health; and stabilizing newborns’ cardiopulmonary system^(28)^.

Despite this, a study that sought to analyze good practices adopted in the care of women and newborns, in a public maternity hospital in Bahia supported by the Stork Network, showed that immediate skin-to-skin contact with the baby was only possible for 51.6% of women^(29)^.

This study observed the guarantee of the principle of “the right to health at the highest level” when a newborn was referred to specialized care and to the Neonatal Intensive Care Unit, as well as multidisciplinary medical care for pregnant women at the time of delivery, referral for examinations and care in the Intensive Care Unit for both, when necessary. The reports show that the care provided to postpartum women met the particularities of SCD, such as the issue of discomfort due to the cold, a situation that was valued and resolved with simple actions described in video 2.

Obstetric violence and respectful maternal care are intertwined, interfacing with the health care model. In the context of collective health, the RMC Charter principles clash with a market-based health model, because in this model, if health care is aimed at profit: providing information to women means wasting time for producing care; allowing fathers to enter the delivery room means wasting surgical equipment and clothing; giving women autonomy to choose what conduct to follow means minimizing the scientific power of health professionals who define the option to follow. In this model, the objective is centered on the disease, not on promoting the health and well-being of these women. Thus, even in a model that focuses on the disease, pregnant women or postpartum women with a chronic disease such as SCD do not receive respectful care, demonstrating that the challenge of assisting women during childbirth, from the perspective of respectful maternal care, is much more complex.

In order to guarantee the “right to healthcare and to the highest attainable level of health”, the narratives highlighted specialized care, the receipt of information, the institution physical structure, access to tests and their interpretation. Thus, the components for respectful maternal care need to be developed and supported by an effective and robust health system in its structure, in addition to interpersonal relationships between women and care providers. A study showed that interventions to promote respectful maternal care, at a global level, are categorized into: individual level, referring to behavioral actions of women, communities and service providers; meso level, resolving gaps in facilities; and macro level, seeking to improve services based on public policies, strategies and guidelines for health systems. Promoting respectful maternal care is a way to attract more users to health services, making their birth narratives more pleasant, and can reduce morbidity and mortality from preventable causes^(30)^.

### Study limitations

It would be possible to gain a deeper understanding of the postpartum women’s experience by including other virtual platforms, such as birth stories described on Instagram^®^. However, YouTube™ is the platform that allows for the inclusion of iconographic analysis. Similarly, netnographers who include virtual interview stages with the owners of videos/posts can relate the findings to sociodemographic characteristics as well as highlight detailed data.

### Contributions to health, nursing or public policy

Based on the reflections of this study, we hope to contribute to some aspects of women’s health care, such as: providing adjustments to the pedagogical projects of undergraduate courses, including the theme of SCD, structural racism and communication skills with patients; deconstructing professional perspectives on vertical relationships and the overvaluation of medical power; encouraging health professionals to boost women’s leading role in childbirth from prenatal care, offering information for empowerment; alerting to the need to reduce stress and anxiety levels of postpartum women, as well as reducing the risk of postpartum sadness (baby blues), linked to the traumatic experience of childbirth; and reflecting on the transgression of the care model progressing from mechanistic, productivist and technicist, in search of interactionist, humanitarian and sustainable care.

## FINAL CONSIDERATIONS

Considering the UN SDGs 2030 that apply to respectful care for postpartum women with SCD, there is a challenge for them to ensure equality in care between black and non-black women as well as empowerment during prenatal care and childbirth.

One gap to be studied was the (in)visibility of other professionals in narratives about the childbirth of women with SCD, since the focus was on the doctor-patient relationship. This study promotes reflections for the implementation of respectful maternal care in the context of collective health care for women with SCD. The current paradigm in health services values productivity and quantification of interventions performed. Consequently, what is best for those involved, from a structural point of view, will not always be best for postpartum women. Care needs to be comprehensive, with qualified listening and directed at resolving the unique demands of postpartum women with SCD.

Given the evidence from this study, in addition to the WHO’s efforts to improve childbirth care, there is an urgent need to mobilize to encourage respectful maternal care in health services, from a broader perspective of this concept that still needs to be analyzed.
