# Supplementary material for: Respectful care for postpartum women with sickle cell disease: a netnographic study
Source: Rev Bras Enferm. 2024 Dec 16;77(6):e20230545. doi: 10.1590/0034-7167-2023-0545 (PMC11654558; doi:10.1590/0034-7167-2023-0545)
Supplement: Translation

# Translation: Cuidado respeitoso às parturientes com doença falciforme: estudo netnográfico

**DOI:** 10.1590/0034-7167-2023-0545pt

**Authors:** Zannety Conceição Silva do Nascimento Souza, Luana Gabriella Pinheiro Barrêto, Paulo Roberto Lima Falcão do Vale, Elysangela Dittz Duarte, Cristiane dos Santos Silva, Evanilda Souza de Santana Carvalho

**Affiliations:** I Universidade Estadual de Feira de Santana. Feira de Santana, Bahia, Brasil; II Hospital Unimed Baía de Todos os Santos. Feira de Santana, Bahia, Brasil; III Universidade Federal do Recôncavo da Bahia. Santo Antônio de Jesus, Bahia, Brasil; IV Universidade Federal de Minas Gerais. Belo Horizonte, Minas Gerais, Brasil

**Keywords:** Parturiente, Doença Falciforme, Cuidado, Enfermagem Obstétrica, Assistência à, Saúde Culturalmente Competente.

## Abstract

**Objetivos:**

analisar os princípios do cuidado materno respeitoso em narrativas de parturientes com doença falciforme, relacionando-os com os Objetivos de Desenvolvimento Sustentável.

**Métodos:**

estudo netnográfico, com dois vídeos publicados em 2020. Análise iconográfica e temática dedutiva pela *Respectful Maternity Care Charter*, organizado no *software* MAXQDA.

**Resultados:**

princípios identificados foram direito: à liberdade contra danos e maus-tratos; à informação, consentimento informado, recusa de procedimentos médicos, respeito pelas escolhas e preferências da mulher incluindo acompanhante; ser considerada pessoa desde seu nascimento, com tratamento digno e respeitoso; saúde no mais alto nível possível; recém-nascido estar com seus pais ou responsáveis. Os Objetivos de Desenvolvimento Sustentável até 2030 para as mulheres não foram contemplados positivamente na experiência das parturientes.

**Considerações Finais:**

é oportuno que trabalhadores da saúde se qualifiquem para exercer o cuidado materno respeitoso, com escuta qualificada, compreensão, resolução das demandas singulares das parturientes com doença falciforme, buscando igualdade no atendimento às mulheres.

## INTRODUÇÃO

A atenção à mulher com doença falciforme (DF) na gestação, parto e puerpério requer visibilidade, por ser uma doença genética e hereditária que, no Brasil, tem estimativa de 60 mil a 100 mil pessoas acometidas. A DF possui relevância clínica e epidemiológica, apresentando elevada prevalência e morbimortalidade, sobretudo na população negra brasileira, sendo considerada, portanto, um importante problema de saúde pública no Brasil^(1,2)^. No Brasil, a maior incidência é apresentada nos estados da Bahia, Distrito Federal e Piauí^(3)^.

No ano de 2001, por meio da Portaria nº 822 do Ministério da Saúde (MS), foi instituído o rastreamento neonatal da DF e outras hemoglobinopatias através do teste do pezinho no Programa Nacional de Triagem Neonatal (PNTN). Em 2011, o MS incluiu a realização do exame eletroforese de hemoglobina na rotina do pré-natal para gestantes e parceiros^(3)^. Essas iniciativas denotam o entendimento da gravidade da DF e a importância de sua detecção precoce.

Entre 2014 e 2020, houve uma média anual de diagnósticos de casos novos de crianças pela triagem neonatal de 1.087, com incidência de 3,78 a cada 10 mil nascidos vivos. Sobre a mortalidade, dados do Sistema de Informações de Mortalidade (SIM), relacionados aos anos de 2014 e 2019, evidenciam maior número de óbitos na faixa etária entre 20 e 29 anos. Entre crianças de 0 a 5 anos de idade, registra-se em média um óbito por semana^(3)^. Esses dados mostram que é necessário dar visibilidade a este problema de saúde pública. Embora não seja exclusivo da população negra, nela se concentra a maioria dos adoecidos, e em razão da maior vulnerabilidade social deste grupo, também se registra o maior índice de mortalidade em consequência da doença^(1-4)^.

A DF interfere na sociabilidade das mulheres, no exercício de sua sexualidade, nas escolhas reprodutivas, nos relacionamentos conjugais, nos projetos de maternidade, nas relações interpessoais e na autoimagem^(5)^. Além disso, a mulher com DF tem sua gestação e parto permeados de expectativas diferentes das não adoecidas. Por ser a gestação considerada de risco, exige frequentes exames, controle rigoroso dos hábitos de vida, envolvendo insegurança quanto à própria saúde e do feto, medo da morte e a iminente necessidade de submeter-se à cesariana^(6)^.

As desigualdades de raça, gênero e classe, níveis de escolaridade e renda, e condições de moradia aprofundam a vulnerabilidade das mulheres negras, quando comparadas às mulheres brancas, interferindo no acesso aos serviços^(7,8)^ e, consequentemente, no prognóstico da doença. Parte-se do pressuposto de que as mulheres com DF possuem singularidades em sua vivência de gestação, trabalho de parto e parto determinadas pelo adoecimento crônico, mas também pelo racismo e iniquidades sociorraciais. Desta forma, o racismo trata-se de “uma forma sistemática de discriminação que tem a raça como fundamento e que se manifesta por meio de práticas conscientes ou inconscientes que culminam em desvantagens ou privilégios para indivíduos a depender do grupo racial ao qual pertençam”^(9)^.

O racismo implica preconceito, discriminação racial e, consequentemente, práticas discriminatórias que resultam, entre outras coisas, em tratamento diferenciado para grupos marcadamente racializados. Em vista desse entendimento, uma das formas de se coibir o racismo na assistência às mulheres negras no ciclo gravídico-puerperal é promovendo um cuidado respeitoso. A promoção do cuidado materno respeitoso é mencionada como um elemento na melhoria das estratégias de cuidado em maternidade, abrangendo o atendimento aos direitos fundamentais da mulher, do recém-nascido e da família, por meio do acesso equitativo aos cuidados baseados em boas práticas^(10)^.

Nessa perspectiva, o setor de saúde pública global ressalta a importância de entender e discutir os danos vividos por mulheres no parto nos serviços de saúde. Em 2011, a *White Ribbon Alliance* publicou a *Respectful Maternity Care (RMC) Charter: The Universal Rights of Women and Newborns*, implementada em muitos países, como uma ação deste movimento global para defender a maternidade segura como prioridade dos governos^(11)^. Em 2014, a Organização Mundial da Saúde (OMS) divulgou uma declaração intitulada *The prevention and elimination of disrespect and abuse during facility-based childbirth*, utilizando *RMC Charter* como uma de suas referências, reafirmando os direitos humanos fundamentais da mulher durante o parto, e destacou o “direito à saúde no mais alto nível possível”, o qual inclui o direito a um cuidado de saúde digno e respeitoso^(12)^.

Respaldado nos direitos das mulheres e recém-nascidos no contexto da gravidez e parto, o cuidado materno respeitoso é apresentado pela *RMC Charter* em dez princípios a serem contemplados durante o cuidado: direito à liberdade contra danos e maus-tratos; direito à informação, consentimento informado, recusa de procedimentos médicos, respeito pelas escolhas e preferências da mulher incluindo acompanhante; direito à privacidade e à confidencialidade; direito a ser considerada pessoa desde seu nascimento, com tratamento digno e respeitoso; direito à igualdade, ausência de discriminação e atenção equitativa; direito à saúde no mais alto nível possível; direito à autodeterminação, autonomia e liberdade contra detenção arbitrária; direito de o recém-nascido estar com seus pais ou responsáveis; direito à identidade e nacionalidade da pessoa desde o nascimento; e direito à alimentação adequada e água potável^(11)^.

Para alcance do parto e nascimento seguros, as políticas públicas no Brasil definiram como diretrizes equidade, respeito, redes de atenção, parto adequado, prevenção à mortalidade materna, pré-natal, prevenção de prematuridade, letramento, empoderamento e engajamento, participação da família e comunidade^(13)^.

Essas diretrizes, bem como os princípios da *RMC Charter*, alinham-se aos Objetivos de Desenvolvimento Sustentável (ODS) até 2030, propostos pela Organização das Nações Unidas (ONU). Dois desses objetivos refletem sobre a saúde das mulheres, quando propõem “garantir o acesso à saúde de qualidade e promover o bem-estar para todos em todas as idades” e “alcançar a igualdade de gênero e empoderar todas as mulheres e meninas”^(14)^.

Diante das iniciativas globais para o parto respeitoso, discutir o cuidado às parturientes com doença crônica convoca-nos a acessar o conhecimento disponível para romper com a determinação social na saúde e a refletir sobre o cuidado ofertado às mulheres negras no ciclo gravídico-puerperal, uma vez que esse grupo está mais vulnerável às violências, negligências e violação de direitos resultantes do racismo nas instituições de saúde.

O racismo se manifesta nas instituições de saúde não somente através do preconceito e da discriminação racial, mas também do racismo institucional (RI), que implica a construção e manutenção de sistemas de desigualdade baseadas na raça/cor da pele dos indivíduos, reproduzido nas instituições, públicas ou privadas. Constitui-se uma dimensão do racismo na qual as pessoas recebem tratamentos desiguais, com resultados desiguais dentro das unidades de saúde, configurando-se uma exclusão seletiva de grupos considerados racialmente subordinados. É, portanto, uma expressão do racismo que ultrapassa a dimensão individual e se instala como uma dimensão estrutural, por meio de políticas, práticas, normas e formas de organização que perpetuam atitudes racistas para as pessoas usuárias do serviço de saúde^(15)^. Assim, não é possível abordar o cuidado à saúde da mulher negra sem discutir também o racismo.

As demandas de saúde da mulher negra, gestante e parturiente com DF ainda carecem de visibilidade tanto nas políticas públicas quanto nas discussões e produções científicas sobre o cuidado respeitoso no parto, visto que não está nítido o que essas mulheres consideram como cuidado respeitoso. Assim, este artigo foi desenvolvido a partir da seguinte questão: quais os princípios do cuidado materno respeitoso identificados nas narrativas de parturientes com DF?

## OBJETIVOS

Analisar os princípios do cuidado materno respeitoso em narrativas de parturientes com DF relacionando-os com os ODS.

## MÉTODOS

### Aspectos Éticos

Sobre os aspectos éticos, foram considerados os fundamentos de respeito à privacidade, honra, inviolabilidade da imagem e dignidade da pessoa, conforme a Lei Geral de Proteção de Dados Pessoais (LGPD), Lei n° 13.709/2018. Destaca-se que os vídeos da plataforma digital são de acesso público gratuito e, em virtude disso, não houve contato direto com as proprietárias do canal. Além disso, não é necessário o consentimento escrito das proprietárias, pois as mesmas enquanto titulares dos dados tornaram as informações públicas^(16)^. Respeitando o sigilo e o anonimato, a identidade e a imagem das parturientes foram omitidas, e os *links* dos vídeos selecionados não foram revelados.

### Tipo de Estudo

Estudo netnográfico, realizado na plataforma de vídeos *YouTube* ^TM^. Este relato de pesquisa qualitativa se pautou no *COnsolidated criteria for REporting Qualitative research* (COREQ)^(17)^. A netnografia investiga fenômenos sociais experienciados na internet mediante uso de aparelhos eletrônicos. É possível utilizar comunicações digitais, investigar interações em redes sociais, *blogs*, fóruns e sites, buscando interpretar como a cultura netnográfica se expressa^(18)^.

### Cenário do estudo

A plataforma digital *YouTube* ^TM^, criada em 2005, permite compartilhamento de vídeos e a visualização por milhões de pessoas que acessam a internet (por isso a escolha deste campo para o estudo), sendo um veículo de informações importante, favorecendo a interação de quem assiste por meio de comentários, *likes* e *dislikes* ^(19)^.

### Fonte dos dados

Os dois vídeos selecionados abordam relatos sobre experiências de parto de duas mulheres que convivem com a DF e possuem algumas características: vídeo 1 - produzido em 2020, após sete meses do parto da relatora, possui duração de 35 minutos e 03 segundos, com 589 visualizações até 18 de abril de 2023; vídeo 2 - produzido também em 2020, após três meses do parto da relatora, possui duração de 5 minutos e 47 segundos, com 2.200 visualizações até 18 de abril de 2023. Sobre ambos, foram preservados os nomes por questões éticas.

### Coleta e organização dos dados

A primeira etapa da coleta e organização dos dados consistiu em conhecer o que as mulheres narravam sobre o parto no contexto da DF. Na segunda etapa, procedeu-se à busca e seleção dos vídeos no *YouTube* ^TM^ incluindo os que abordam experiências de parto vivenciadas por mulheres com “DF” ou “anemia falciforme”, sem limite de data de postagem, no idioma português do contexto brasileiro, contendo experiências narradas pelas próprias mulheres. Foram excluídos vídeos com conteúdo pejorativo, de entretenimento ou duvidosos (*fake news*). Para verificar a veracidade do conteúdo dos vídeos, utilizamos a SCAN^(20)^.

Para a construção da estratégia de busca, os autores 1, 3 e 6 elaboraram o protocolo com as seguintes palavras-chave (*tags*): experiência; “parto normal”; “anemia falciforme”. Em seguida, procedeu-se à busca piloto na caixa de pesquisa do *YouTube* ^™^. Uma das *tags* foi “parto normal”, por perceber que nos levaria a vídeos sobre partos, mas não necessariamente este foi um critério de inclusão, visto que o parto cesáreo é frequente em mulheres com DF. Para evitar viés de seleção dos vídeos, essa etapa foi realizada às cegas pelos autores 1, 2 e 3 (qualificados no método). Mediante ausência de consenso, a autora 6 (*expert* no método e temática) contribuiu para a decisão final. Os dados foram organizados em documento do *software Microsoft Word^®^*, versão 16. O protocolo completo da coleta de dados, que inclui as etapas de identificação, elegibilidade e inclusão, em atendimento ao fluxograma *Preferred Reporting Items for Systematic Reviews and Meta-Analyses* (PRISMA)^(21)^, está ilustrado na Figura 1.

**Figura 1:** Constituição de *corpus* adaptado da proposta PRISMA^(21)^

A amostragem dos vídeos foi intencional, sendo selecionados pela riqueza de informação que se buscava, envolvendo a reflexividade e a compreensão em profundidade dos significados subjetivos naquele contexto cultural^(22)^.

Na terceira etapa, os vídeos selecionados foram assistidos, e os depoimentos das mulheres foram transcritos na íntegra por uma quarta pesquisadora independente (autora 2). A transcrição foi validada pela autora 1. Na quarta etapa, o texto transcrito foi organizado em *Microsoft Word^®^* em arquivos individuais e inseridos no *software* MAXQDA, versão 20 (número de referência 282394887). Na quinta etapa, foi construído o plano de análise iconográfica.

Na sexta etapa, organizou-se a lista de códigos com dicionário dos significados respaldada pela *RMC Charter* ^(11)^, a qual foi discutida e consensuada pela equipe de pesquisa para guiar a codificação.

Na sétima etapa, procedeu-se à codificação pela autora 1, que tinha experiência na temática, e pela autora 4, com *expertise* na pesquisa qualitativa. Visando à confiabilidade na análise, a codificação ocorreu de forma independente pelas autoras 1 e 2, guiada pela lista de códigos e com suporte do MAXQDA. Ainda nesta fase, em paralelo à codificação por pares, aconteceu a análise iconográfica dos vídeos, pela autora 2 e autor 3, de forma independente, e posterior validação por toda a equipe de pesquisa. Com isso, a coleta e a análise seguiram um processo de triangulação de pesquisadores.

Na etapa oito, resumo narrativo foi elaborado para descrever os resultados encontrados. Na etapa nove, adotou-se a triangulação dos dados analisados, sendo eles obtidos na codificação (temas atribuídos às transcrições) dos trechos das narrativas correspondentes e imagens, conforme descrito no Quadro 1.

**Quadro 1:** Princípios da *Respectful Maternity Care Charter*, narrativas relacionadas e análise iconográfica das imagens dos vídeos do *YouTube* ^TM^

Princípio da *RMC Charter* ^*^ (código)	Narrativa selecionada	Análise iconográfica	Inferência
Direito à liberdade contra danos e maus-tratos^▲^	*Ah... depois de todo esse erro dela, dela não saber o que é anemia falciforme e querer me mandar para o clínico... ela teve o segundo erro... dela não ver meu raio x. Eu fiquei internada durante uma semana, sentindo bastante cansaço, sabe... eu chorava porque não tava aguentando mais… a.... médica viu o jeito que eu tava tudo e falou assim: “Você tem que aguentar o máximo que der... quanto mais o seu bebê ficar aí dentro, vai ser melhor...”* (V1 - minuto inicial 6’59’’ até 7’40”).	Descrição V1: 8”30’ Pálpebras se distanciam, abrindo os olhos ao máximo. Lábios se aproximam, permanecendo uma pequena fenda entre eles, com arcada dentaria superior cerrada sobre inferior. Cantos da boca se alongam, aproximando-os das orelhas. Ambas as mãos tocam as bochechas de cada lado da face simultaneamente. O terceiro quirodáctilo de cada mão toca a ponta final da sobrancelha do seu respectivo lado, região palmar próximo ao mento.	Expressões de susto e apreensão ao relatar a experiência de atendimento médico.
Direito de a criança estar com seus pais ou responsáveis^▲^	*...eles perguntaram: “Doutor, pode mostrar o bebê para ela?”, eu não sei porque, sabe? ... eu não sei se realmente eles achavam que eu não ia sobreviver... Então, eles me mostraram ela de relance, já colocaram ela na incubadora e levaram para UTI neonatal, porque ela nasceu prematuro... eu não lembro dos traços, como ela nasceu... e ela nasceu com dois quilos e meio, agora dos centímetros... eu não sei até hoje. Eu voltei para UTI, eu não pude ver ela mais... até depois que eu pude ter ela nos meus braços... pude ir para casa... tive alta junto com ela...* (V2 - minuto inicial 4’13” até 5’25’).	Descrição V2: 5’ 21” Cabeça baixa, olhando para baixo, na direção do bebê que se encontra em seu colo (sendo possível visualizar apenas uma pequena parte do couro cabeludo desta). Lábios aproximados, fenda entre eles expondo arcada dentária superior, contração do músculo risório e zigomático, linhas de expressão em bochechas.	Angústia pela espera da filha. Alegria e satisfação por estar em casa com sua filha.
Direito a ser considerada pessoa desde seu nascimento, com tratamento digno e respeitoso^▲^	*Aí eu lembro que chegou um médico... ridículo, sabe? Eu queria saber que medicação eu tava tomando, o que eu tinha, queria saber tudo!... ele pegou, olhou para minha cara e fez: “Aí, você não tá aqui porque você tá com pneumonia, você tá aqui por causa da sua anemia!”* (faz expressão de ironia) ... *acredita que ele falou isso pra mim? A minha anemia não tava baixa... dores... essas coisas da anemia eu não tava sentindo nada... a única coisa que eu estava sentindo era falta de ar, cansaço, fraqueza, sabe? E dor no pulmão, que isso eu sentia, então tudo que eu tava sentindo era pela pneumonia, e ele olhou assim para minha cara e disse que não era pela pneumonia, era por causa da anemia... então, nossa! Queria dar um murro na cara daquele médico... veio falar comigo: “Os teu exame tá todo alterado e você vai ter que ficar, no mínimo, mais setes dias aqui, porque seu exame deu que você tá com uma... uma infecção que... já tá no... seu corpo inteiro”. Ele falou desse jeito para mim: “E a gente não sabe que infecção que é essa, então você tem que ficar aqui para gente mudar seu remédio por um remédio bem mais forte”. Eu já ficando nervosa, chateada, porque como eu ia ficar mais setes dias longe da minha filha? Aí eu já comecei a chorar, comecei desistir...* (V1 - minuto inicial 26’13” até 29’14”).	Descrição V1: 28’ 41” Mulher aproxima as pálpebras com força, bem como as sobrancelhas. Os dedos das mãos se aproximam em pares na frente da face, região lateral dos dedos indicadores, tocando o espaço entre sobrancelhas. As palmas das mãos estão afastadas, e o 1º quirodáctilo aponta para fora da face.	Nervosismo, tristeza, indignação, irritação e agressividade dirigida ao médico por não responder às suas perguntas sobre seu estado de saúde com clareza. Desespero, mal estar, perda de apetite, repercussões da má comunicação de notícias difíceis e sofrimento por distanciamento da filha recém-nascida.
Direito à informação, consentimento informado, recusa de procedimentos médicos, respeito pelas escolhas e preferências da mulher incluindo acompanhante^▲^	*... falaram: “... a gente vai ver se marca seu parto... pra amanhã!”. Tinha acabado de acordar, ainda não tinha tomado café, depois voltaram para mim e falaram assim: “Oh, a gente vai fazer seu parto...”. Aí tive que correr, ligar para o meu marido, ligar para minha mãe. Não foi exatamente a hora que eles falaram, atrasou um pouco o parto. Enfim, foram me buscar e me levaram lá para sala de cirurgia. Como meu caso era mais grave, eu tive que fazer a cesárea, não tive escolha...* (V2 - minutos 0’47” até 1’57”).	Descrição V2: 1’ 33” Mulher lateraliza levemente a cabeça para a esquerda, vira os olhos para lado esquerdo e para cima, contrai lábios e músculo risório. Levanta a mão esquerda, com a superfície palmar vidara para cima e os cincos quirodáctilos afastados uns dos outros.	Expressão irônica de surpresa e insatisfação.
Direito à saúde no mais alto nível possível**^■^**	*... na sala, tinha a equipe que ia fazer o meu parto, obstetras, médicos dos meus problemas de saúde, médico da UTI, hematologista, que eu tenho anemia falciforme. Eu gostei muito, porque eles desligaram o ar condicionado, colocaram um colchão embaixo da maca para me aquecer, porque quem tem anemia falciforme sabe como é que é. Teve um anjo* (anestesista)*... sempre ia me falando: “A gente vai fazer tal procedimento... essa injeção é para essa coisa...”. Como eu tava sentindo muita falta de ar, ele também me ajudou, ele aumentava o ar. Quando eu já tava ok e a minha saturação tava ok, ele abaixava mais um pouquinho* (V2 - minuto 1’33’’ até 2’34”).	Descrição V2: 1’ 33’’ Expressão facial séria, gesticula com as mãos enquanto relata. Pisca bastante os olhos, movimenta suavemente a cabeça e tronco durante o relato, sorri ao falar que desligaram o ar condicionado.	Relaxamento e conforto ao mencionar práticas de cuidado, como desligar o ar condicionado.

*Legenda:*

▲ *direitos negados;* ^**■**^direitos assegurados;

* RMC - Respectful Maternity Care.

### Análise dos dados

Para a análise dos dados, utilizou-se a análise iconográfica, que possibilita examinar e interpretar conteúdos visuais a partir dos detalhes selecionados nestes. Além disso, é possível comparar imagens, atribuindo um sentido e significado ao contexto, às formas como imagens e grupos distintos se relacionam, e discutindo-as com o subsídio de textos, arquivos, entrevistas e afins^(23)^.

A análise iconográfica ocorreu em quatro momentos^(24)^: 1) visualização na íntegra dos vídeos selecionados, sem áudio e sem legenda, buscando as expressões que estivessem relacionadas ao fenômeno estudado, com registro (*print*); 2) visualização com pausas, sem áudio e legenda, análise de imagens estáticas, orientada pelas seguintes questões: quem são os atores? Quais os objetos inanimados e outras características dos ambientes que compõem cada cena? 3) visualização dos vídeos com pausas, áudio e legendas, com o objetivo de responder às seguintes questões: quais as expressões corporais que se destacam? Quais os símbolos, gestos e comportamentos que ilustram as experiências vivenciadas? Como interagem os atores? Quais os princípios do cuidado respeitoso identificados? 4) visualização dos vídeos na íntegra, sem pausas, com áudio e legenda.

Na análise temática, foram observados os elementos que respondiam à questão de pesquisa, padrões de significado amplos. Após revisão dos temas, refinou-se e definiu-se o padrão de significados. Na escrita final dos resultados e análise, relacionou-se a narrativa aos dados extraídos e à literatura existente^(25)^. Essa análise foi temática dedutiva, pois foi guiada pelo interesse teórico nos princípios da *RMC Charter*, ao analisar as narrativas das mulheres. Assim, a partir de um construto teórico existente, o fenômeno foi analisado de forma detalhada nos aspectos dos dados que se relacionavam aos princípios citados^(25)^.

## RESULTADOS

## DISCUSSÃO

As narrativas das mulheres com DF estudadas evidenciaram aspectos de cinco princípios defendidos na *RMC Charter* para o cuidado respeitoso em maternidade, porém a maioria deles possui fragilidades no que tange à garantia dos direitos. Além disso, é possível perceber que o estabelecido como ODS até 2030 não foi contemplado positivamente na experiência das parturientes.

No que se refere aos ODS^(14)^, o ODS 3 apresenta a meta de “Assegurar uma vida saudável e promover o bem-estar para todas e todos, em todas as idades”. No item 3.1 desse ODS, é preconizado, até 2030, a redução da mortalidade materna em todo o mundo para valor menor que 70 mortes por 1000.000 nascidos vivos, e no item 3.2, aborda-se que é necessário acabar com as mortes evitáveis de recém-nascidos e crianças menores de 5 anos (redução pelo menos de 25 por 1.000 nascidos vivos), com todos os países buscando a redução da mortalidade neonatal para pelo menos 12 por 1.000 nascidos vivos.

Os erros e equívocos nas habilidades dos profissionais de saúde no acompanhamento das parturientes com DF, evidenciados nas falas das mesmas, fragilizam o alcance desse ODS, visto que uma formação profissional em saúde que não contempla conteúdos e práticas de cuidado às mulheres com essa singularidade pode ocasionar complicações do quadro de saúde dessas e dos fetos, aumentando o risco de mortalidade materna, fetal ou neonatal.

Os princípios da *RMC Charter*, como “direito à liberdade contra danos e maus-tratos” e “direito à informação”, não foram garantidos em sua totalidade; houve orientação sobre a necessidade de manutenção da gestação pelo maior tempo possível devido ao risco ao feto, mas essa informação não foi clara para que a mulher entendesse que se relacionava ao risco de parto prematuro. À medida que a mulher com DF expressou sua dor decorrente da fisiopatologia da doença, a resposta do profissional se restringiu a que esta suportasse os desconfortos para manter a gestação e beneficiar o feto. Mulheres negras recebem menos orientações sobre a gestação, parto, pós-parto e situações de emergência nas consultas de pré-natal, quando comparadas às mulheres brancas, sendo ainda muito restritas e fragmentadas, com menos qualidade à medida que a escolaridade da mulher diminui^(26)^.

Ainda que a gestação seja um evento potencialmente grave para as mulheres com DF, essa condição não se constitui impedimento para a geração de filhos, sendo de suma importância que, durante a gestação, a mulher seja devidamente orientada pelos profissionais da equipe multidisciplinar, recebendo informações sobre os riscos e possíveis complicações de sua gestação, parto e pós-parto.

Assim, pode-se refletir sobre o que seriam causas dessa baixa oferta de informações: formação pouco humanitária, mecanicista, produtivista e intervencionista dos profissionais de saúde; maior valorização das intervenções tecnológicas aos corpos humanos; neutralidade emocional na relação com as pessoas atendidas; e racismo estrutural.

A relação vertical e unilateral e a supervalorização do conhecimento científico e epistemicídio do saber popular provocado pelos profissionais de saúde restringem o acesso, dificultando a formação de vínculo e, consequentemente, o cuidado em uma perspectiva longitudinal. Esses aspectos infringem o item 3.7^(14)^, pois esse trata de assegurar o acesso universal aos serviços de saúde sexual e reprodutiva, incluindo planejamento familiar, informação e educação, o que foi negado pelas falas das relatoras.

No relato do vídeo 1, evidencia-se uma descontinuidade da assistência prestada na instituição e despreparo dos profissionais de saúde. Nesse contexto, sobre o atendimento às mulheres gestantes com DF, o MS aponta que “os profissionais de saúde estão ainda muito despreparados para atuar no cuidado, em particular durante a gestação, o que pode contribuir para aumentar a insegurança e o medo que elas experimentam nessa fase da vida”^(6)^.

O despreparo é expresso no lidar com o risco gestacional, na falta de articulação das diversas instituições que são procuradas pelas mulheres com DF em busca de cuidado, e nesta pesquisa, especificamente na conduta, que demonstra uma preocupação maior com a restrição do crescimento fetal, que pode induzir a um parto prematuro, pela frase “segurar a gestação ao máximo que puder”, e outras com pouco entendimento da mulher, como “infecção que tá no corpo inteiro”, pelo relato de demora de diagnóstico de pneumonia, mesmo tendo feito raio-X, descrito no vídeo 1. A pneumonia é outra condição muito comum na pessoa com DF. Assim, a expressão “mais alto nível possível de saúde” inclui não somente o acesso a equipamentos e serviços diagnósticos, mas também a assistência de profissionais qualificados.

A análise iconográfica do vídeo 1 demonstra nervosismo, tristeza, indignação, irritação e agressividade dirigida ao médico, por não responder às perguntas da mulher sobre seu estado de saúde com clareza. Isso cabe uma reflexão de como o pouco conhecimento e o despreparo profissional para lidar com a DF podem contribuir para um cuidado desumanizado e desrespeitoso para a reprodução de violências e violação de direitos de mulheres negras com DF, e isso também pode implicar desfechos negativos para a saúde da mãe e do bebê.

O fato de a mulher relatar condutas inadequadas, descrever seus questionamentos para os profissionais de saúde, às vezes de forma irônica, pode deixar a impressão de que esta utilizou empoderamento. Estudo realizado com mulheres no Nepal^(27)^ concluiu que houve associação significativa entre empoderamento das mulheres e a utilização de serviços de saúde materno. O estudo categorizou o empoderamento em três elementos ((1) da mídia e da tecnologia da informação; (2) econômico; e (3) sociocultural e familiar/interpessoal), sendo os dois primeiros mais influentes do que o último nessa busca por serviços qualificados. Para os pesquisadores, em relação às mulheres “... proporcionar e incentivá-las a se tornarem financeiramente independentes e o acesso à mídia e à tecnologia da informação pode aumentar o número de mulheres que procuram serviços de saúde materna qualificados”.

Mas ao se pensar na assistência ao parto da mulher negra com DF, que possui vulnerabilidade por diversas questões já mencionadas, com seu parto sendo definido como cesáreo, sem a possibilidade de escolha, esse empoderamento ainda é um objetivo almejado que precisa ser alcançado através das ações dos profissionais, das instituições e das políticas públicas. O poder dos profissionais de saúde ainda está imposto e culturalmente estabelecido nos serviços de saúde, em detrimento ao protagonismo das mulheres, inclusive pela formação dos profissionais que alimenta esse poder.

Alcançar a igualdade de gênero e empoderar todas as mulheres e meninas, acabando com todas as formas de discriminação que compõem o ODS 5, transversaliza essa situação no parto de mulheres com DF, e é um dos mais difíceis objetivos a serem alcançados, pois parte da cultura do poder do saber científico se sobrepõe à vontade feminina, seu conhecimento e autonomia sobre seu corpo. É importante aumentar o uso de tecnologias de informação e comunicação, com políticas sólidas para o empoderamento.

O cuidado respeitoso em maternidade também abrange o recém-nascido e o seu direito de estar com seus pais ou responsáveis. Esse cuidado foi observado neste estudo, com pontos divergentes, como: as mulheres relataram que não foram apresentadas ao recém-nascido logo após o parto, o que suscita a preocupação de uma necessidade de assistência médica de emergência ou se houve uma atitude de descuidado nesse sentido.

É importante avaliar que o contexto e as condições clínicas do recém-nascido podem exigir cuidados intensivos que impedem a mãe de vê-lo imediatamente após o parto. Em situações similares a essa, a equipe necessita explicar a necessidade do afastamento temporário entre mãe e recém-nascido para cuidados imediatos e comunicar as possibilidades terapêuticas, reduzindo estresse e ansiedade e garantindo segurança de mãe e filho. O contato pele a pele busca estimular um contato precoce do recém-nascido com a mãe ao nascer e, sempre que possível, deve ser realizado ainda na sala de parto, pois possui muitos benefícios para ambos: criação de vínculo; aumento da tranquilidade do recém-nascido; diminuição da perda de temperatura corporal, do risco de hipoglicemia e do tempo de hospitalização; estímulo ao aleitamento materno e descida do leite na primeira meia hora de vida; redução da ansiedade da mãe para confirmar a segurança e saúde do filho; e estabilização cardiopulmonar do recém-nascido^(28)^.

Apesar disso, estudo que buscou analisar as boas práticas adotadas na atenção à mulher e ao recém-nascido, em uma maternidade pública baiana apoiada pela Rede Cegonha, evidenciou que o contato pele a pele imediato com o bebê foi possibilitado apenas para 51,6% das mulheres^(29)^.

Observaram-se, neste estudo, a garantia do princípio “direito à saúde no mais alto nível”, quando se encaminhou o recém-nascido a cuidados especializados e à Unidade de Terapia Intensiva Neonatal, bem como pela assistência médica multidisciplinar à gestante no momento do parto, a referência de realização de exames e a assistência em Unidade de Terapia Intensiva para as duas, quando foi necessário. Os relatos mostram que o cuidado à parturiente atendeu às particularidades da DF, como a questão do desconforto pelo frio, situação valorizada e resolvida com ações simples descritas no vídeo 2.

A violência obstétrica e o cuidado materno respeitoso estão entrelaçados, fazendo interface com o modelo de atenção à saúde. No âmbito da saúde coletiva, os princípios da *RMC Charter* confrontam-se com um modelo mercadológico da saúde, pois, nesse modelo, se o cuidado em saúde visa ao lucro: dar informações para a mulher é perder tempo de produção de cuidado; deixar o genitor entrar na sala de parto é desperdício de roupa e aparatos cirúrgicos; dar autonomia à mulher para ela escolher qual a conduta a ser seguida é minimizar o poder científico do profissional de saúde que define a opção a seguir. Nesse modelo, o objetivo é centrado na doença, não na promoção da saúde e do bem-estar dessas mulheres. Assim, mesmo em um modelo que enfoca a doença, mulheres grávidas ou parturientes com uma doença crônica como a DF não alcançaram cuidados respeitosos, demonstrando que o desafio da assistência à mulher no parto, na perspectiva de cuidado materno respeitoso, é bem mais complexo.

No sentido de garantir o “direito à saúde no mais alto nível de saúde possível”, as narrativas evidenciam a assistência especializada, o recebimento de informações, a estrutura física da instituição, o acesso a exames e a interpretação desses. Assim, os componentes para cuidado materno respeitoso precisam ser desenvolvidos e amparados por um sistema de saúde eficaz e robusto em sua estrutura, além das relações interpessoais entre mulher e provedor de cuidados. Estudo mostrou que intervenções para a promoção do cuidado materno respeitoso, em nível global, são categorizadas em: nível individual, referindo-se a ações comportamentais de mulheres, comunidades e prestadores de serviços; nível meso, resolvendo lacunas em instalações; e nível macro, buscando a melhoria dos serviços a partir de políticas públicas, estratégias e diretrizes para os sistemas de saúde. Promover cuidado materno respeitoso é uma forma de atrair mais usuárias aos serviços de saúde, tornando suas narrativas de parto mais agradáveis, podendo reduzir a morbimortalidade por causa evitáveis^(30)^.

### Limitações do estudo

O aprofundamento da experiência de parturientes seria possível a partir da inclusão de outras plataformas virtuais, como os relatos de parto descritos no *Instagram* ^®^. Entretanto, o *YouTube* ^TM^ é a plataforma que permite a inclusão da análise iconográfica. Da mesma forma, netnógrafos que incluam etapas de entrevistas virtuais com as proprietárias dos vídeos/postagens podem relacionar os achados às características sociodemográficas, bem como evidenciar dados minuciosos.

### Contribuições para as áreas da saúde, enfermagem ou políticas públicas

A partir das reflexões deste estudo, espera-se contribuir para alguns aspectos da atenção à saúde da mulher como: providenciar ajustes nos projetos pedagógicos dos cursos de graduação, incluindo a temática da DF, racismo estrutural e habilidades de comunicação com os pacientes; desconstruir perspectivas profissionais acerca das relações verticais e supervalorização do poder médico; incentivar profissionais de saúde a estimular o protagonismo da mulher no parto desde o pré-natal, ofertando informações para o empoderamento; alertar para a necessidade de diminuição nos níveis de estresse e ansiedade das parturientes, bem como redução do risco da tristeza pós-parto (*baby blues*), vinculados à experiência traumática do parto; e refletir sobre a transgressão do modelo de cuidado progredindo do mecanicista, produtivista e tecnicista, em busca do cuidado interacionista, humanitário e sustentável.

## CONSIDERAÇÕES FINAIS

Considerando os ODS 2030 da ONU que se aplicam ao cuidado respeitoso à parturiente com DF, existe um desafio para que esses consigam garantir a igualdade na assistência entre as mulheres negras e não negras, bem como o empoderamento durante o pré-natal e parto.

Uma lacuna a ser estudada foi a (in)visibilidade dos demais profissionais nas narrativas sobre o parto de mulheres com DF, visto que o enfoque foi a relação médico-paciente. Este estudo promove reflexões para a implementação do cuidado materno respeitoso no contexto da atenção à saúde coletiva de mulheres com DF. O paradigma vigente nos serviços de saúde valoriza a produtividade e a quantificação das intervenções realizadas. Consequentemente, o que é melhor para os envolvidos, do ponto de vista estrutural, nem sempre será o melhor para a parturiente. O atendimento precisa ser compreensivo, com escuta qualificada e direcionado à resolução das demandas singulares das parturientes com DF.

Diante das evidências deste estudo, somando-se aos esforços da OMS em qualificar a atenção ao parto, urge uma mobilização para incentivo do cuidado materno respeitoso nos serviços de saúde, em uma perspectiva mais ampla deste conceito que ainda precisa ser analisado.
